# Mesenchymal Stem Cells Promote the Osteogenesis in Collagen-Induced Arthritic Mice through the Inhibition of TNF-*α*

**DOI:** 10.1155/2018/4069032

**Published:** 2018-05-08

**Authors:** Chang Liu, Huayong Zhang, Xiaojun Tang, Ruihai Feng, Genhong Yao, Weiwei Chen, Wenchao Li, Jun Liang, Xuebing Feng, Lingyun Sun

**Affiliations:** ^1^Department of Rheumatology and Immunology, The Affiliated Drum Tower Hospital, Nanjing University Medical School, 321 Zhongshan Road, Nanjing 210008, China; ^2^Department of Rheumatology and Immunology, Dalian Municipal Central Hospital, 826 Southwest Road, Dalian 116033, China

## Abstract

**Objective:**

To investigate the effects of umbilical cord mesenchymal stem cell (UC-MSC) transplantation on joint damage and osteoporosis in collagen-induced arthritis (CIA) mice and to explore the mechanisms by which UC-MSCs modulate the osteogenic differentiation.

**Methods:**

CIA mice were divided into the following treated groups: UC-MSC transplantation group, antitumor necrosis factor- (TNF-) *α* group, and zoledronic acid (ZA) group. Microcomputed tomography (micro-CT) was used to analyze the bone morphology parameters. Osteogenic differentiation of treated CIA mice was determined. Bone marrow mesenchymal stem cells (BM-MSCs) from CIA mice were treated with TNF-*α* in vitro to explore their effects on osteogenesis.

**Results:**

The arthritis score was significantly reduced in the UC-MSC transplantation and anti-TNF-*α*-treated CIA groups, compared with control mice (*P* < 0.001). Micro-CT showed that CIA mice developed osteoporosis at 12 weeks after immunization. The bone morphology parameters were partially improved in UC-MSC-treated CIA mice. Impaired osteogenic differentiation functions were indicated by decreased ALP activity (*P* < 0.001) and reduced mRNA and protein levels of osteogenic marker genes (*P* < 0.05) in CIA mice compared with DBA/1 mice. UC-MSC treatment significantly upregulated the impaired osteogenic differentiation ability in CIA mice. Meanwhile, the serum TNF-*α* level was decreased significantly in the UC-MSC group. The osteogenesis was reduced with the addition of TNF-*α* in vitro.

**Conclusion:**

This study demonstrated that UC-MSC transplantation not only significantly improved the joint damage but also played a beneficial role in osteoporosis in CIA mice. Mechanistically, the improved osteogenic differentiation of CIA under UC-MSC treatment may be achieved by inhibition of TNF-*α*.

## 1. Introduction

Rheumatoid arthritis (RA) is an autoimmune disease in which chronic inflammation causes severe damage to the joints. Several types of skeletal manifestations are present in RA, including bone erosions, periarticular osteopenia, and generalized osteoporosis. The prevalence of osteoporosis in RA is higher when compared to age-matched controls and can become a severe comorbidity [1]. Patients affected by RA, mainly those with high disease activity, have a twofold risk of developing osteoporosis compared to the general population and almost the double risk for hip and vertebral fractures [2]. This secondary osteoporosis is one of the main reasons of bone structural deterioration, caused by multifactorial factors including estrogen deficiency, long-term glucocorticoid therapy, and systemic inflammation [3].

Bone remodeling depends on a functional balance among osteoclasts and osteoblasts. In RA, proinflammatory cytokines, including tumor necrosis factor- (TNF-) *α*, interleukin- (IL-) 1, IL-6, and IL-17, disturb the balance between osteoclast and osteoblast activity, often resulting in a net loss of bone. TNF-*α* has been implicated as an important mediator of inflammation and joint destruction in RA. Many studies showed that TNF-*α* plays a central role in promoting osteoclast activity [4], although recent studies also showed that TNF-*α* regulates the activity of osteoblasts, which play an important role in bone metabolism together with osteoclasts [5]. Majority of studies of RA osteoporosis were focusing on the overactivated osteoclasts, while few paid attention to osteoblast regulation and the underlying mechanisms. Therefore, in the present study, one of our focuses is on the regulation of osteoblast differentiation in RA.

Mesenchymal stem cells (MSCs) isolated from bone marrow (BM), umbilical cord (UC), or adipose tissue are multipotent progenitor cells, capable of differentiating into tissue-forming cells, such as bone and cartilage. Many studies confirmed that MSCs could differentiate into osteoblasts when isolated and cultured in vitro [6]. Besides their multilineage differentiation potential, MSCs also have immunosuppressive activities owing to their paracrine effects and interactions with different immune cells. These properties of MSCs therefore offer a new strategy in the treatment of numerous autoimmune diseases and demonstrate promising results in safety and efficacy. Our previous studies showed that allogeneic MSCs have extraordinarily therapeutic effects on patients with refractory lupus, inflammatory bowel disease (IBD), RA, and polymyositis/dermatomyositis (PM/DM) [7–11]. In RA patients with osteoporosis, we also observed an increased bone mineral density (BMD) after transplantation (unpublished). However, the mechanisms that mediated the beneficial effects of UC-MSCs in RA patients, such as prevention of osteoporosis, remain unclear.

We hypothesized that xenogeneic MSC transplantation could promote the differentiation of autologous BM-MSCs into osteoblasts in a mouse model of collagen-induced arthritis (CIA). In this study, we investigated the effect of UC-MSC transplantation on joint damage and systemic osteoporosis in CIA mice and explored the mechanisms by which UC-MSCs modulate the osteogenic differentiation. The present study will provide novel mechanisms of applying MSC treatment in patients with RA.

## 2. Materials and Methods

### 2.1. Animals

Eight-week-old male DBA/1 mice which have been established as a stable CIA model [12, 13] were obtained from Shanghai SLAC Laboratory Animal Co. Ltd. All mice were housed in a specific pathogen-free environment under controlled conditions. All animal procedures were approved by the institutional animal care and use committee of the Affiliated Drum Tower Hospital of Nanjing University Medical School.

### 2.2. Induction of CIA

CIA models were induced by immunization on day 1 and day 21, respectively, as described previously [14]. Bovine type II collagen (CII; Sigma, USA) was dissolved in acetic acid at 4°C overnight. Subsequently, collagen was emulsified 1 : 1 with complete Freund's adjuvant (CFA; Sigma, USA) or incomplete Freund's adjuvant (IFA; Sigma, USA), yielding a final concentration of 1 mg/ml. CIA was induced in each animal by intradermal injection of emulsified collagen in CFA or IFA over the base of the tail (100 *μ*l/mouse). Clinical arthritis scores were assigned on a scale of 0~4 (0 = no swelling or erythema, 1 = erythema and mild swelling confined to the midfoot (tarsals) or ankle joint, 2 = erythema and mild swelling extending from the ankle to the midfoot, 3 = erythema and mild swelling extending from the ankle to metatarsal joints, and 4 = erythema and severe swelling encompassing the ankle, foot, and digits) [15]. The individual mouse arthritis score was obtained by summing the scores recorded for each limb, with a maximum score of 16. The severity of arthritis in each mouse was determined independently and blindly by two investigators, and the mean of the two scores was calculated.

### 2.3. Treatment of CIA

Treatment was initiated after the onset of disease (on day 28 after the first immunization), when arthritis had become established (arthritis score ≥ 2). All the treatments were administered intraperitoneally. CIA mice were randomly assigned to four groups (*n* = 8/group). In the UC-MSC transplantation group, UC-MSCs of 5 × 10^6^ cells were injected intraperitoneally twice, on day 28 and day 56 after the first immunization, respectively. In the anti-TNF-*α*-treated group, anti-TNF-*α* (eBioscience, USA) was administrated at a total dose of 200 *μ*g/mouse, for 50 *μ*g/mouse every two weeks. The zoledronic acid (ZA, Chiatai Tianqing, China) group was treated with 150 *μ*g/kg ZA for once. The control group was treated with PBS. All animals were sacrificed after initial treatment for 8 weeks.

### 2.4. MSC Culturing

UC-MSCs were isolated and cultured from fresh UCs obtained from two natural deliveries in the Affiliated Drum Tower Hospital of Nanjing University Medical School. The study protocol was approved by the ethics committee of the above institute. Written informed consent was obtained from all donors. The UCs were rinsed twice in PBS consisting of penicillin and streptomycin to remove the cord blood. Then the washed cords were cut into 1 mm^2^ pieces and floated in DMEM-F12 containing 10% FBS (Stemcell, Vancouver, Canada). The pieces of cord were subsequently incubated at 37°C in a humidified atmosphere containing 5% CO_2_. The medium was replaced every 3 days after the initial plating. When well-developed colonies of fibroblast-like cells appeared after 10 days, the cultures were trypsinized and passaged for further expansion. Flow cytometric analysis confirmed that the cells expressed CD73, CD90, and CD105, but not CD14, CD34, CD45, CD79, or HLA-DR. The capacity of MSCs to differentiate along adipogenic and osteogenic lineages was evaluated. UC-MSCs at passages 3 with a purity of more than 95% were used.

BM-MSCs were isolated and cultured from the bone marrow of DBA/1 or treated CIA mice. The bone marrow cells harvested from long bones were seeded into 25 cm^2^ culture flasks with a density of 1 × 10^5^ cells/cm^2^, cultured for 3-4 days with DMEM-F12 containing 20% FBS, and incubated at 37°C in a humidified atmosphere containing 5% CO_2_. The adherent cells were cultured continually, and the culture medium was changed every 3 days. After reaching 80% confluence (after 7–10 days), the cultures were trypsinized and passaged into a new flask for further expansion. The cells were identified as described above. BM-MSCs at passage 3 were used for follow-up differentiation experiment.

### 2.5. Micro-CT

Computed tomographic images of the right femur in all groups (*n* = 3) were acquired before treatment endpoint, using a micro-CT Scan SkyScan1176S scanner at a resolution of 9 *μ*m. For verification of bone structure, 3-dimensional models of the femur were reconstructed using SkyScan CT Analyzer version 1.8. BMD (mg/cm^2^), trabecular bone volume/total volume (BV/TV, %), trabecular thickness (Tb.Th, *μ*m), trabecular number (Tb.N, 1/mm), and trabecular space (Tb.Sp, mm) were assessed by scanner software (Skyscan CTAn).

### 2.6. Histopathology

4% paraformaldehyde-fixed limbs were decalcified and paraffin embedded using standard histologic techniques. Tissue sections were prepared and stained with haematoxylin and eosin (H&E) to assess the histological scores. The extent of synovial inflammation and bone/cartilage destruction was determined using a 0~4 scale (0 = normal synovium, 1 = synovial membrane hypertrophy and cell infiltrates, 2 = pannus and cartilage erosion, 3 = major erosion of cartilage and subchondral bone, and 4 = loss of joint integrity and ankylosis) according to the previous study [16].

### 2.7. In Vitro Osteogenesis

After reaching 80% confluence, BM-MSCs at passage 3 were cultured in osteogenic induction medium (R&D, USA) for 7~14 days as different experiments needed. The medium was changed every 3 days. Alkaline phosphatase (ALP) staining and ALP activity were determined using NBT/BCIP (Beyotime, China) and ALP detection kit (Beyotime, China) according to the manufacturer's protocol.

### 2.8. Real-Time Quantitative Reverse Transcription PCR (qRT-PCR)

Total RNA of osteoblasts was extracted with the TRIzol reagent (Takara, Japan). Reverse transcription was performed using a PrimeScript RT reagent kit (Takara, Japan). The real-time PCR reactions were carried out using the QuantiTect SYBR Green PCR kit (Takara, Japan) and the Applied Biosystems 7500 Real-Time PCR System following the manufacturer's protocol (Applied Biosystems, USA). All samples were run in triplicates, and cDNA levels were normalized to those for GAPDH. The sequences of primers used are shown in Table 1.

### 2.9. Western Blot

BM-MSCs at passage 3 were cultured in osteogenic induction medium for 7 days. Then the adherent cells were directly lysed in buffer containing phosphatase inhibitor (RIPA protein lysate 1 ml, phosphatase/proteinase inhibitor 10 *μ*l, phenylmethylsulfonylfluoride (PMSF) 10 *μ*l) at 4°C for 30 minutes. The lysates were homogenized, denatured at 99°C for 10 min and centrifuged at 4°C for 10 min. Samples were fractioned by 10% SDS-PAGE gel and transferred onto a PVDF membrane. The membranes were blocked with 5% milk in Tris-buffered saline buffer (0.05% Tween20) for 1 h and incubated at 4°C overnight with the following primary antibodies: the osteogenic markers ALP (R&D Systems, USA), Osterix (Abcam, Cambridge, England), and *β*-actin (Immunoway, USA). Primary antibodies were used at 1 : 1000 dilution, and a secondary horseradish peroxidase-conjugated anti-rabbit IgG antibody was used at 1 : 5000 dilution. The blots were visualized using an enhanced chemiluminescence kit (SYNGENE, England) according to the manufacturer's instructions.

### 2.10. Measurement of TNF-*α* Levels in Serum

TNF-*α* in serum was measured by ELISA using the mouse TNF-*α* assay kits (eBioscience, USA) according to the manufacturer's protocol.

### 2.11. Statistical Analysis

All data were expressed as mean ± SEM. Differences between groups were evaluated by one-way analysis of variance followed by post hoc Tukey's multiple comparison tests. *P* values < 0.05 were considered to be significant. Analyses and graphical representation were performed using GraphPad Prism™ software (GraphPad).

## 3. Results

### 3.1. UC-MSC Transplantation Improved Arthritis of CIA

**Figure 1:**
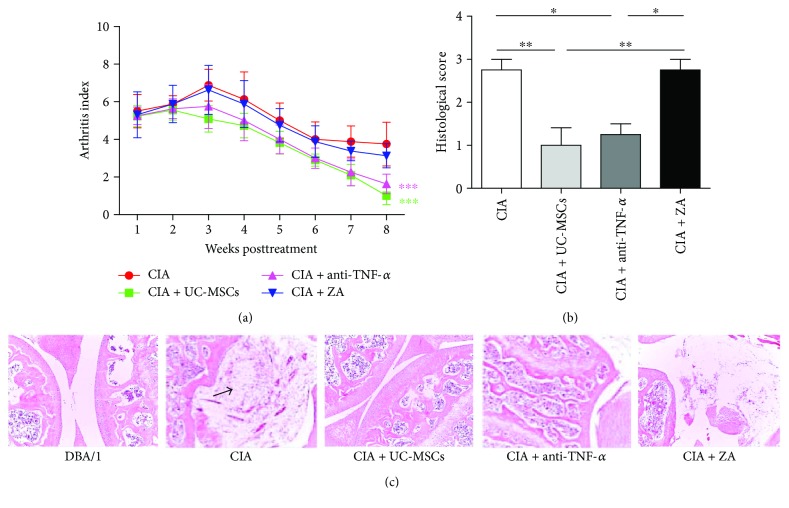
UC-MSCs alleviated the severity of arthritis and histological scores, comparable to TNF-*α* inhibitor treatment. (a) Arthritis scores in the collagen-induced arthritis (CIA) groups with different treatments (*n* = 8 in each group). CIA mice were treated with PBS (control), antitumor necrosis factor- (TNF-) *α*, zoledronic acid (ZA), and umbilical cord mesenchymal stem cells (UC-MSCs). (b) The evaluation of histological scores of H&E-stained sections in different groups (*n* = 4 in each group). (c) H&E-stained sagittal sections of knee joints from CIA mice (photographed at ×100). Arrow indicates fibrinoid necrosis. ^∗^*P* < 0.05; ^∗∗^*P* < 0.01; ^∗∗∗^*P* < 0.001.

CIA models were successfully set up on day 28 post initial immunization. CIA mice were divided into four groups, including the CIA control group, the UC-MSC transplantation group, the anti-TNF-*α*-treated group, and the ZA-treated group. All mice were treated for 8 weeks. We evaluated the arthritis score of CIA mice every week. The pathological manifestation of knee joints was evaluated by H&E staining. As shown in Figure 1(a), the arthritis score was significantly reduced in the UC-MSC transplantation and the anti-TNF-*α*-treated groups compared with control mice (*P* < 0.001). There was no significant difference in the arthritis score between ZA-treated and control mice. Consistent with the results of arthritis score, the pathological score of knee joint was significantly reduced in the UC-MSC transplantation group (*P* < 0.01) and the anti-TNF-*α*-treated group (*P* < 0.05) compared with the control and ZA group (Figure 1(b)). In Figure 1(c), DBA/1 mice showed an entire joint surface and no synovial hyperplasia, inflammatory cell infiltration, or bone erosion; however, the joint of CIA mice presented a serious synovial hyperplasia, inflammatory cell infiltration, and even fibrinoid necrosis.

### 3.2. UC-MSC Transplantation Played a Beneficial Role in CIA Osteoporosis

**Figure 2:**
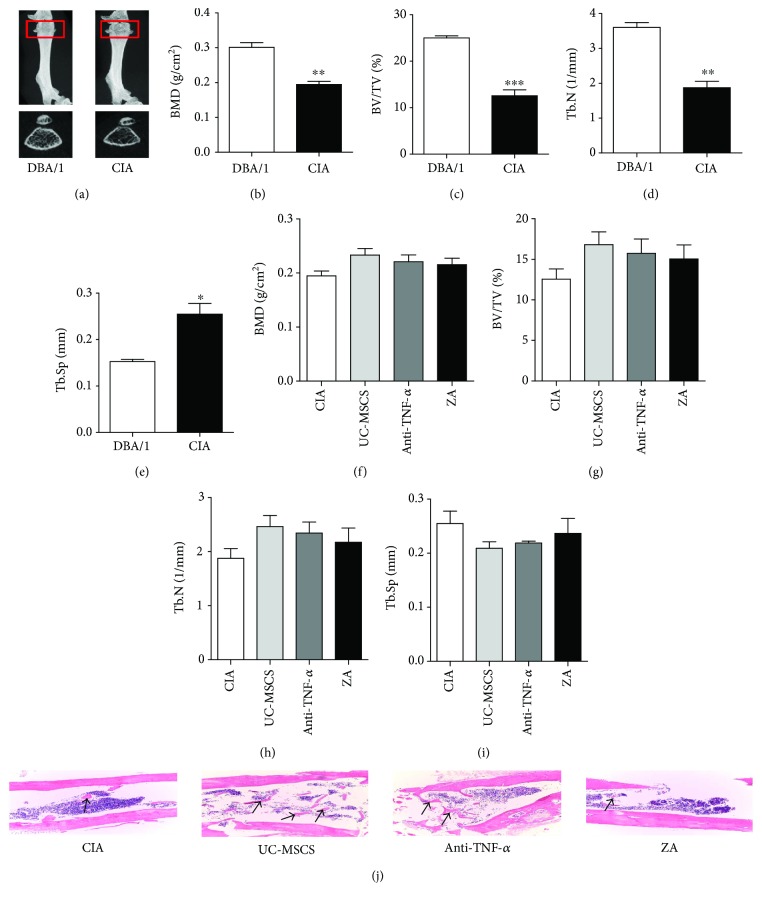
UC-MSC transplantation played a beneficial role in osteoporosis in CIA mice. (a–e) Micro-CT showed that the bone mineral density (BMD), trabecular bone volume/total volume (BV/TV), and trabecular number (Tb.N) of the femur were significantly decreased and trabecular separation (Tb.Sp) was significantly increased in CIA mice, compared with that in DBA/1 mice (*n* = 3 in each group). (f–i) The BMD (*P* = 0.0659), trabecular BV/TV (*P* = 0.1046), Tb.N, and Tb.Sp were partially improved in UC-MSC-treated CIA mice compared with control mice (*n* = 3 in each group). (j) H&E staining showed an increased Tb.N (arrow) in UC-MSC-treated CIA mice than in the control and ZA group (photographed at ×100). ^∗^*P* < 0.05; ^∗∗^*P* < 0.01; ^∗∗∗^*P* < 0.001.

To identify whether bone loss occurred in CIA mice, micro-CT was used to analyze the bone morphology parameters at the end point.  Figure 2 showed that the bone mass in the image (Figure 2(a)), BMD (*P* < 0.01) (Figure 2(b)), trabecular bone volume/total volume (BV/TV) (*P* < 0.001) (Figure 2(c)), and trabecular number (Tb.N) (*P* < 0.01) (Figure 2(d)) of the femur were significantly decreased in CIA mice, compared with those in DBA/1 mice, and the trabecular space (Tb.Sp) was significantly increased in CIA mice (*P* < 0.05) (Figure 2(e)). After treatment for 8 weeks, micro-CT showed that BMD (*P* = 0.0659) (Figure 2(f)), trabecular BV/TV (*P* = 0.1046) (Figure 2(g)), Tb.N (Figure 2(h)), and Tb.Sp (Figure 2(i)) were partially improved in UC-MSC-treated mice compared with control mice. H&E staining also showed an increased Tb.N in UC-MSC-treated mice than in the control and ZA group (Figure 2(j)).

### 3.3. UC-MSC Treatment Significantly Upregulated the Impaired Osteogenic Differentiation Ability In Vitro

**Figure 3:**
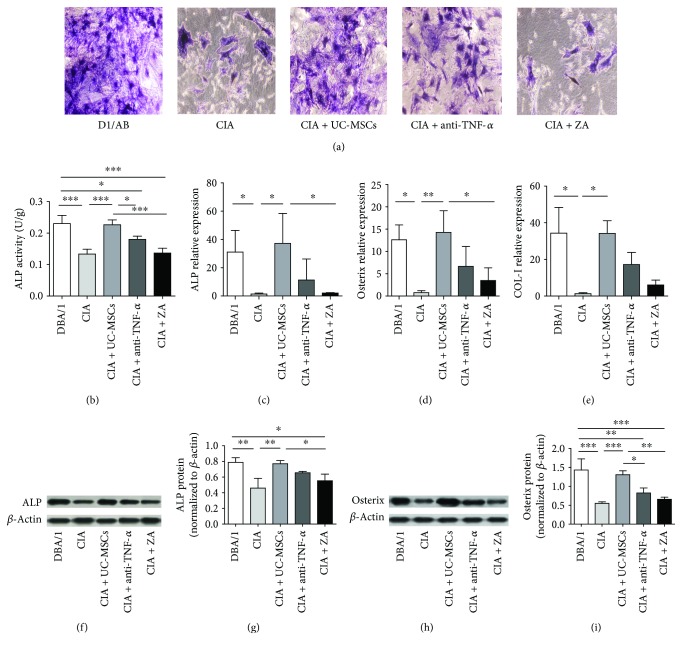
UC-MSC treatment significantly improved the impaired osteogenic differentiation ability *in vitro*. (a) Representative images showing ALP staining in intact (DBA/1), the CIA groups with no treatment, UC-MSC treated, anti-TNF-*α*, and zoledronic acid (ZA) treatment (photographed at ×100). (b) ALP activity was significantly deduced in the CIA group, and UC-MSC transplantation significantly increased ALP activity (*n* = 4 in each group). (c–e) UC-MSC transplantation significantly upregulated the mRNA levels of osteogenic genes including ALP, Osterix, and COL-I. (f–i) UC-MSC treatment significantly upregulated the ALP and Osterix protein levels in CIA mice. ^∗^*P* < 0.05; ^∗∗^*P* < 0.01; ^∗∗∗^*P* < 0.001.

To determine the osteogenic differentiation ability of various groups, autologous BM-MSCs derived from treated CIA mice and DBA/1 mice were cultured in the osteogenic medium. Osteogenic differentiation was determined by ALP staining and ALP activity on day 7. The expression of the osteogenic markers ALP, Osterix, and COL-I was analyzed by qRT-PCR on day 7 and day 14, respectively.  Figure 3(a) showed decreased ALP-positive cells in the CIA group than in the DBA/1 group and increased ALP-positive cells in the UC-MSC group. Consistent with the general morphology, ALP activity was significantly reduced in the CIA group than in the DBA/1 group (*P* < 0.001), and UC-MSC transplantation significantly increased ALP activity compared with CIA controls (*P* < 0.001) (Figure 3(b)). Similarly, the expression levels of osteogenic genes ALP, Osterix, and COL-I were significantly downregulated in the CIA group than in the DBA/1 group (*P* < 0.05). UC-MSC transplantation significantly upregulated the mRNA levels of ALP (*P* < 0.05), Osterix (*P* < 0.01), and COL-I (*P* < 0.05) than CIA control (Figures 3(c)–3(e)). Anti-TNF-*α* treatment tended to upregulate the expression of osteogenic genes, but there was no significant difference compared with CIA control. As shown in Figures 3(f)–3(i), UC-MSC treatment significantly upregulated the ALP (*P* < 0.01) and Osterix (*P* < 0.001) protein levels in CIA mice.

### 3.4. Improved Osteogenic Differentiation of CIA under UC-MSC Treatment May Be Related to Inhibition of TNF-*α*

**Figure 4:**
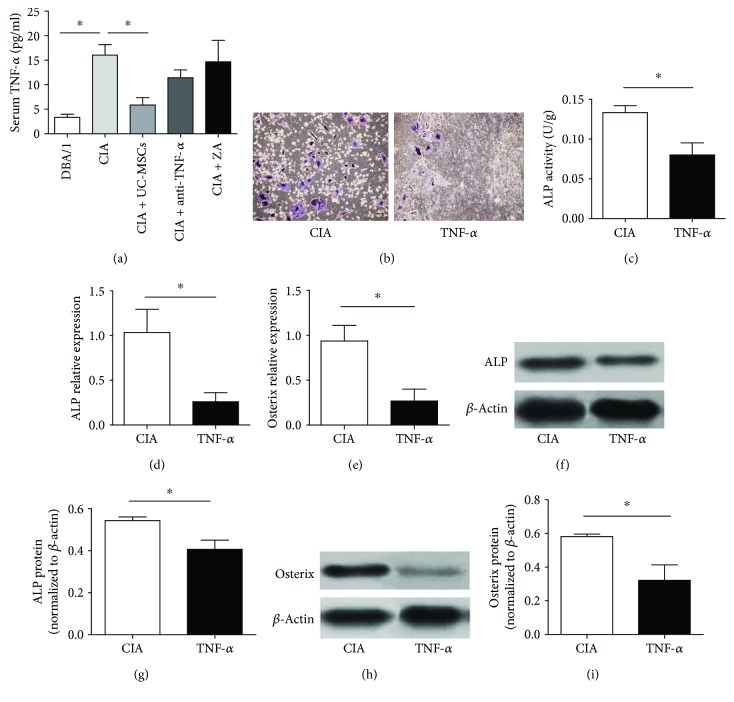
Improved osteogenic differentiation of CIA under UC-MSC treatment may be related to inhibition of TNF-*α*. (a) The serum TNF-*α* level was significantly decreased in the UC-MSC group (*n* = 8 in each group). (b) Representative images showing ALP staining in CIA without or with TNF-*α* treatment. (c) ALP activity was significantly decreased in the TNF-*α* addition group. (d–i) The expression levels of osteogenic genes and protein levels of ALP and Osterix were significantly downregulated in the TNF-*α* addition group (*n* = 3 in each group). ^∗^*P* < 0.05.

To identify the possible mechanism by which UC-MSCs promote the osteogenic differentiation of CIA, we analyzed the serum TNF-*α* levels in four groups after 8 weeks. TNF-*α* level was significantly decreased in the UC-MSC group (Figure 4(a)), compared with that in CIA mice. To explore the role of TNF-*α* in osteogenic differentiation in vitro, BM-MSCs derived from CIA mice were cultured in osteoblast differentiation medium in the presence or absence of TNF-*α* (PeproTech, USA) at a concentration of 10 ng/ml for 7 days.  Figure 4(b) showed reduced ALP-positive cells in the TNF-*α*-treated group. Consistent with the general morphology, ALP activity was significantly decreased in the TNF-*α*-treated group when compared with the control group (*P* < 0.05) (Figure 4(c)). The mRNA levels of osteogenic genes and protein levels of ALP and Osterix were significantly downregulated in the TNF-*α*-treated group than in the control group (*P* < 0.05) (Figures 4(d)–4(g)).

## 4. Discussion

The mechanisms of RA-induced secondary osteoporosis are largely unknown. In this study, we used CIA mice as the osteoporosis model. CIA is a classical autoimmune model which resembled RA in many ways. For example, synovitis and erosions of cartilage and bone are hallmarks of both RA and CIA. In our micro-CT scanning, we found that CIA mice developed osteoporosis at 12 weeks after immunization. The BMD, BV/TV, and Tb.N of the femur were significantly decreased in CIA mice, compared with those in DBA/1 mice. As shown in previous study, systemic osteopenia was identified in CIA and adjuvant-induced arthritis (AIA) rats within 20 days of disease onset [17]. Wu et al. identified that BMD of the femur and lumbar vertebrae and biomechanical properties of the femur were decreased in CIA rats. Trabecular BV of the tibia and lumbar vertebrae was decreased whereas bone resorption was increased in CIA rats. Bone formation of the tibial shaft in periosteal surfaces was decreased in CIA rats. Furthermore, the bone loss in CIA rats was more severe at 16 weeks than at 8 weeks [18]. Generally for a CIA mouse model, the peak of arthritis has been demonstrated on days 35 to 42 after the first immunization, and then it tends to enter into the period of remission [13]. In this study, osteoporosis was observed in CIA mice even though the AI was decreased after initial three weeks. Together, these studies along with ours demonstrate that CIA mice can be an appropriate model to study secondary osteoporosis.

In this study, we identified that UC-MSC transplantation not only significantly improved the joint damage but also played a beneficial role in osteoporosis in CIA mice. The bone morphology parameters were partially improved in UC-MSC-treated CIA mice. MSCs are multipotent stromal cells capable of differentiating into different cell lineages including osteoblasts, chondrocytes, and adipocytes [19]. In addition to the differentiation potential, their immune-suppressive properties by modulating T and B cell proliferation and differentiation as well as dendritic cell maturation have garnered increasing attention [20–22]. In our previous study, xenogeneic MSC transplantation can improve CIA arthritis and reduce joint destruction [23], and refractory RA patients exhibited a remission of symptom by decreasing ESR, DAS28, and VAS score after allogeneic MSC transplantation [10]. A larger sample of clinical study also confirmed the safety and efficacy of MSC transplantation in RA patients [24].

To date, studies using MSCs to treat bone structural deterioration mainly focused on estrogen deficiency-induced osteoporosis. Wang et al. identified that transplantation of MSCs can help to strengthen osteoporotic bone in rabbits. After 8 weeks of implantation of autologous MSCs into the ovariectomy (OVX) rabbits, the trabecular thickness and newly formed osteoids were significantly enhanced [25]. In another study, BM-MSCs isolated from healthy rats were injected into the femurs of OVX rats. The results revealed decreased differentiation capabilities of osteoporotic BM-MSCs as compared to healthy controls. Two months after injection, the trabecular bone percentage was improved to a comparable level as in control healthy rats [26]. Yu et al. also identified that allogeneic BM-MSCs might play a critical role in treating estrogen deficiency-induced osteoporosis [27].

Our study showed that the osteogenic differentiation ability of BM-MSCs in CIA mice was impaired, while this ability was promoted under UC-MSC treatment. Many studies have confirmed that MSCs can differentiate into osteoblasts after being isolated and cultured in vitro [28], and induction of osteoblasts from BM-MSCs has been widely used as a method to investigate osteogenic differentiation. BM-MSCs are increasingly being used for many clinical applications, such as orthopedic and reconstructive surgery. Recently, researchers have realized the importance of MSCs in anabolic processes in bone and begun to fundamentally study the mechanisms of abnormal MSCs leading to bone loss and osteoporosis [29]. Studies have shown that proinflammatory cytokines, including TNF-*α*, interleukin- (IL-) 1, IL-6, and IL-17, in RA patients or CIA model play a pivotal role not only in inducing bone resorption but also in contributing to bone loss by direct inhibition of osteoblast differentiation.

In this study, we found that the serum TNF-*α* level was significantly decreased in the UC-MSC treatment group. To further confirm whether the suppression of osteogenesis in CIA MSCs was influenced by TNF-*α*, we treated BM-MSCs from CIA mice with TNF-*α* in vitro to explore its effects on osteogenesis. Results showed that osteogenesis reduced with the addition of TNF-*α*, further suggesting that TNF-*α* plays an important role in suppressing osteogenesis. TNF-*α* is reported to be involved in osteoblast differentiation, maturation, and mineralization. For example, TNF-*α* can inhibit osteoblast differentiation from pluripotent progenitor cells by decreasing Runx2 expression [30]. TNF-*α* potently suppresses Smad signaling induced by TGF-beta and BMP-2 and inhibited mineralization of osteoblasts [31]. In general, SMAD1/5/8 and ERK1/2 are able to activate Runx2 expression. In the subsequent study, we would explore which osteogenic signaling pathway is involved. In addition, TNF-*α* also induces apoptosis in osteoblasts, as well as the production of other proinflammatory cytokines such as IL-1*β* [32, 33]. In contrast, some studies demonstrated that a low concentration of TNF-*α* can enhance and accelerate the differentiation of MSCs towards an osteoblast phenotype [34]. However, the mechanisms involved in bone remodeling and responsible for the “paradoxical effects” of TNF-*α* remain unclear [35]. Combined UC-MSC and TNF-*α* set will help to further clarify the mechanism of UC-MSC on osteoporosis in CIA.

Allogeneic MSCs were reported to decrease the levels of proinflammatory cytokines by mitigating lymphocyte dysfunction in RA. One of our previous studies found that UC-MSCs can suppress proinflammatory cytokine by secreting soluble factors. For example, the high cadherin-11 level by fibroblast-like synoviocytes (FLS) of RA can be suppressed by coculturing with UC-MSCs. IDO, HGF, and IL-10 secreted by UC-MSCs were upregulated, while the inhibitory effect of UC-MSCs was abolished by suppression of IL-10 activity [36]. In addition, allogeneic MSCs could suppress T follicular helper (Tfh) cell differentiation in RA patients partly via the production of indoleamine 2,3-dioxygenase (IDO) [23]. A recent study showed that UC-MSCs can decrease serum levels of IL-1*β* and IL-6 in CIA mice by inhibiting the generation of IL-17^+^ or IFN*γ*^+^ T cells [37]. Future studies will focus on the specific mechanisms by which UC-MSCs downregulate TNF-*α*.

## 5. Conclusion

The present study demonstrated that CIA mice developed osteoporosis at 12 weeks after immunization. UC-MSC transplantation not only significantly improved the joint damage but also played a beneficial role in osteoporosis in CIA mice. Mechanistically, the improved osteogenic differentiation of CIA under UC-MSC treatment may be achieved by inhibition of TNF-*α*.

## Figures and Tables

**Table 1:** Primer sequences used for polymerase chain reaction amplifications.

Gene	Primer nucleotide sequences	
mGAPDH	Forward	5′-TGGCCTTCCGTGTTCCTAC-3′
	Reverse	5′-GAGTTGCTGTTGAAGTCGCA-3′
mALP	Forward	5′-TCCCCATGTGATGGCGTATG-3′
	Reverse	5′-GAAGTTGCCTGGACCTCTCC-3′
mOsterix	Forward	5′-CGATTCCCCCTGAGCTTTGT-3′
	Reverse	5′-CCCATTGGACTTCCCCCTTC-3′
mCOL-I	Forward	5′-TTCTCCTGGCAAAGACGGAC-3′
	Reverse	5′-CGGCCACCATCTTGAGACTT-3′
